# Effects of early exercise training following severe burn injury: a randomized controlled trial

**DOI:** 10.1093/burnst/tkae005

**Published:** 2024-05-07

**Authors:** David R Schieffelers, Tianfeng Ru, Haonan Dai, Ziqing Ye, Eric van Breda, Ulrike Van Daele, Weiguo Xie, Jun Wu

**Affiliations:** Multidisciplinary Metabolic Research Unit (M^2^RUN), MOVANT Research Group, Department of Rehabilitation Sciences and Physiotherapy, Faculty of Medicine and Health Sciences, University of Antwerp, Universiteitsplein 1, 2610 Antwerp, Belgium; Department of Burns, Tongren Hospital of Wuhan University & Wuhan Third Hospital, 241 Peng Liuyang Road, 430060 Wuhan, China; Department of Burns, Tongren Hospital of Wuhan University & Wuhan Third Hospital, 241 Peng Liuyang Road, 430060 Wuhan, China; Department of Burns, Tongren Hospital of Wuhan University & Wuhan Third Hospital, 241 Peng Liuyang Road, 430060 Wuhan, China; Multidisciplinary Metabolic Research Unit (M^2^RUN), MOVANT Research Group, Department of Rehabilitation Sciences and Physiotherapy, Faculty of Medicine and Health Sciences, University of Antwerp, Universiteitsplein 1, 2610 Antwerp, Belgium; Multidisciplinary Metabolic Research Unit (M^2^RUN), MOVANT Research Group, Department of Rehabilitation Sciences and Physiotherapy, Faculty of Medicine and Health Sciences, University of Antwerp, Universiteitsplein 1, 2610 Antwerp, Belgium; OSCARE, Organization for burns, scar after-care and research, Van Roiestraat 18, 2170 Antwerp, Belgium; Department of Burns, Tongren Hospital of Wuhan University & Wuhan Third Hospital, 241 Peng Liuyang Road, 430060 Wuhan, China; Department of Burn and Plastic Surgery, The First Affiliated Hospital of Shenzhen University, Room No. 6-1, Building No. 9, 3002 Sungang West Road, Futian District, 518025 Shenzhen, Guangdong Province, China

**Keywords:** Burns, Exercise, Rehabilitation, Muscle wasting, Cachexia

## Abstract

**Background:**

Despite being a stable component of burn rehabilitation at later stages of recovery, exercise training is not commonly provided during the acute phase of burns. A lack of evidence surrounding its efficacy and safety in severely burned adults has hampered its implementation in acute burn care. The aim of this study was to investigate the capacity of early exercise training to modulate parameters of postburn muscle wasting and quality of life.

**Methods:**

Adults <65 years of age with burns ≥40% total burn surface area (TBSA) were randomly allocated to either receive early exercise (n = 29) in addition to standard care or standard care alone (n = 29). Early exercise involved resistance and aerobic training, which commenced as early as possible and lasted for a duration of 6 to 12 weeks, in line with burn center length of stay. Ultrasound-derived quadriceps muscle layer thickness (QMLT) and rectus femoris cross-sectional area (RF-CSA), lower limb muscle force, Eurocol Quality of Life-5 Dimensions and Burn Specific Health Scale Brief (BSHS-B) were assessed 6 and 12 weeks after baseline. Mixed models were fitted to compare between-group changes over time.

**Results:**

A total of 58 adults [42 (95% confidence interval 40–45) years old; 40–94% TBSA range, 86% previously mechanically ventilated] participated in this study. Exercise commenced 7 days [IQR (interquartile range) 5–9] after burn center admission with an attendance rate of 93%. Allocation to the exercise group had a protective effect on the loss of muscle size from baseline to 6 weeks of follow-up (QMLT: β-coefficient: 0.05 cm, *p* = 0.010; RF-CSA: β-coefficient: 0.05 cm^2^, *p* = 0.045), and resulted in an improved recovery from 6 to 12 weeks (QMLT: β-coefficient: 0.04 cm, *p* = 0.01; RF-CSA: β-coefficient: 0.06 cm^2^, *p* < 0.001). Muscle force increased significantly more in the exercise group than in the control group (β-coefficient: 3.102 N, *p* < 0.001) between 6 and 12 weeks. Besides a marginally significant effect for the BSHS-B domains ‘affect’ and ‘interpersonal relationships’ between 6 and 12 weeks, no benefits were observed in the other assessed quality-of-life measures. No serious adverse events were reported in the exercise group.

**Conclusions:**

The results of this study support the use of early exercise training as a feasible and efficacious therapeutic strategy to manage burn-related changes in muscle size and strength in adults with acute severe burn injury.

Highlights

- Exercise training was administered to severely burned adults during the acute phase.
- Exercise on top of usual care resulted in an improved retention and recovery of muscle size and muscle strength.
- Early exercise training is a safe and efficacious strategy to manage muscle wasting in severely burned adults.

## Background

Postburn muscle wasting is rooted in a combination of hypermetabolism, hyperglycemia, hypercatabolism, inflammation and physical inactivity, all of which are most pronounced during burn center stay [1–6]. There has been a growing awareness of postburn muscle wasting and its potential to increase the disease burden for burn patients from the first days of hospital admission to long after hospital discharge [2,7–11]. In the short term, muscle wasting is associated with impaired wound healing, increased risk of infection, intensive care unit-acquired weakness and difficulty weaning off mechanical ventilation, which might ultimately lead to a protracted hospital length of stay and a delayed recovery [12–15]. Beyond the short-term, potential sequelae of muscle wasting include an increased risk of musculoskeletal, cardiovascular and metabolic morbidity years after the trauma, threatening the survivor’s complete recovery and long-term quality of life [16–20]. Despite its impact, muscle wasting is not consistently used as an intervention target in burn care [21]. At the same time, there has been a growing trend towards exercise commenced during burn center stay, echoed by practice guidelines recommendations [22–27], likely following the long-standing evidence in the wider critical care population. It is during this early phase that exercise would be expected to have the largest preventative effect to counter the underlying processes of postburn muscle wasting [28]. In particular, resistance and aerobic forms of exercise, as anabolic, anti-hyperglycemic and anti-inflammatory stimuli, are used effectively in many other health and disease states, including critical illness, to counteract muscle wasting and associated outcomes [29–32]. In the burn population, preliminary evidence of exercise-induced improvements in inflammation, glycemic control and markers of muscle metabolism have been reported [33–37]. Readers are referred to a recent review by Dombrecht *et al*. that provides an overview of mechanisms by which early exercise training might modulate muscle metabolism [28]. As part of the current practice of burn care, however, resistance and aerobic exercise are rehabilitation components that are often reserved for a time when they are perceived as safer and more comfortable for burn patients, and when there is less interference from open wounds, pain and grafting surgery [21]. Recent evidence supporting the inclusion of resistance and/or aerobic exercise during the acute phase in adult burn patients is based on studies with predominantly non-severe burn injuries [33,38]. However, it is the severe burn population with the highest risk of muscle wasting in whom exercise training during the acute phase of burns could be most beneficial, yet, also in whom its efficacy in adult patients has not been studied as a counteracting strategy. Previous investigations of exercise during the acute phase in this population have focused on other relevant outcomes, and have reported several positive effects, including a shorter length of stay in the ICU and hospital, shorter time to independent walking, greater joint range of motion and fewer scar contractures [39–41]. Minimizing postburn muscle wasting in this population is an important outcome, as postburn muscle wasting impacts the host’s vital metabolic reserve that is integral to sustaining the immune response and overcoming critical illness during the acute phase of burns [8,42–44]. For this reason, a deeper understanding of the efficacy of exercise training commenced early during burn center stay in severely burned adults is needed to explore its role in the management of muscle wasting as a part of inpatient rehabilitation. Therefore, the present study was initiated to test the effects of resistance and aerobic exercise administered during the acute phase on muscle wasting and health-related quality of life during burn center stay of severely burned adults.

## Methods

### Study design

This study was designed as a prospective participant-blinded single-center randomized controlled trial including two arms, with the control group following standard-of-care treatment and the experimental group additionally undergoing a protocolized exercise program as part their burn center stay. The study was registered at US National Institutes of Health (ClinicalTrials.gov) #NCT04372550 and approved by the Ethics Committee of the Wuhan Third Hospital on 10 May 2019 [#QT2019–002].

### Participants

Study participants were recruited at the burn center of the Wuhan Third Hospital, Wuhan, China between December 2019 and November 2022 after being screened for eligibility upon admission. Adults were deemed eligible if they were between 18 and 64 years of age at the time of admission and presented with severe burn injuries ≥40% total burn surface area (TBSA). The rationale to exclude adults >64 years of age was taken in view of the differing metabolic responses that adult burn patients >64 and <65 years of age have been shown to exert [45,46], while acknowledging that such an age cutoff does not represent a physiological cut-off point. Exclusion criteria for participation comprised electrical burns, palliative care, pregnancy, lower limb fractures or amputations, or any premorbid neurological, cardiovascular or psychological disorders expected to interfere with the intervention or outcome assessment. As soon as testing was available, all participants were tested for a SARS-COV2 infection prior to burn center admission and during burn center stay. While this was not a formal exclusion criterion, burn patients with a SARS-COV2 infection were not admitted to the burn center to avoid cross-contamination. Written informed consent was obtained from all participants or their next-of-kin in line with the declaration of Helsinki.

### Standard-of-care

Standard-of-care treatment was provided to all participants and entailed standard intensive care, wound care, surgical care, nutritional care, positioning and physical therapy. Physical therapy consisted of passive and active range of motion exercises, stretching, splinting and compression garments. Under the standard-of-care, the scalp (if available) was used as a standard donor site for grafting, and strict post-grafting immobilization was applied for 3–5 days in case of split-thickness autografts. Standard feeding regimens on the ICU were based on energy requirements as calculated with the Peng equation [47], with protein content set at 1.5–2.0 g/kg/day. Participants on the ward received food ordered from the hospital canteen or outside sources. The standard glycemic target for patients requiring exogenous insulin administration was set at 144–180 mg/dl. As the study took place throughout the COVID-19 pandemic, visits by family were prohibited throughout the entire hospital stay.

### Early exercise training

**Figure 1:**
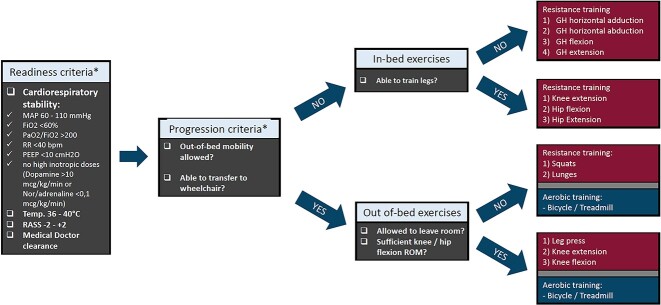
Decision-making tree to guide choice of exercise. *MAP* mean arterial pressure, *FiO_2_* inspired oxygen fraction, *PaO_2_/FiO_2_* arterial oxygenation relative to inspired oxygen, *RR* respiratory rate, *bpm* breaths/min, *PEEP* positive end expiratory pressure, *RASS* Richmond Agitation Sedation Scale, *ROM* range of motion, *GH* glenohumeral. *Criteria to be checked prior to each training session

In addition to the standard-of-care treatment, participants allocated to the exercise group underwent an inpatient 6- to 12-week-long exercise training program consisting of resistance and aerobic training. All exercises were administered by physiotherapy staff trained in the management of burns. The exercise program took place at the bedside in the burn ICU unit and burn ward or using designated exercise equipment in the rehabilitation unit of the burn center. Participants had to meet predefined readiness criteria before beginning the exercise program to ensure medical safety and feasibility of the exercise intervention. These readiness criteria were assessed prior to each exercise session and encompassed parameters of cardiorespiratory stability, body temperature, alertness and cooperation in line with international safety recommendations of active exercise during critical illness [48]. The primary goal of the exercise program was to counteract muscle wasting. Hence, exercises that primarily targeted the lower limbs were prioritized, as collectively these include the largest amount of muscle mass in the body. Resistance training was provided at a frequency of three sessions per week at a volume of three exercises consisting of three sets of 8 to 12 repetitions each. Exercises progressed from in-bed to out-of-bed exercises with free weights or strength appliances depending on individual mobility status (Figure 1). In-bed resistance exercises were provided at a target intensity of 60% of the peak force as produced during a maximal voluntary contraction using a hand-held dynamometer (for methods see the ‘muscle force’ section) (Lafayette, IN, USA), while the intensity of out-of-bed exercises was determined using 8-repetition maximum testing [49]. Aerobic training was carried out twice weekly on a bicycle ergometer or a treadmill, with sessions entailing 24 min of interval training consisting of alternating 3-min bouts of 50 or 70% of peak wattage or metabolic equivalents [50], as assessed by a maximal ramp test. The steep ramp test provided peak wattage values for cycling, as described by De Backer *et al*. [51], while the Naughton protocol or the modified Bruce protocol was used on the treadmill for patients with or without walking impairment, respectively. The exercise program was built and progressed based on general principles of strength and cardiorespiratory conditioning [52,53], with the relative exercise intensity maintained by weekly repetition of the respective maximal tests.

### Outcomes

Assessment of muscle size, muscle force and quality of life was conducted throughout hospital stay at baseline, 6 weeks after baseline, and if participants had not been discharged yet, additionally at 12 weeks. On the day before follow-up assessment, the intensity of the experimental exercise program was reduced to prevent interference with the outcome assessment. The assessment was carried out by a team of three assessors, who were trained prior to the study to ensure uniformity. Results of previous assessments were not checked during follow-up assessment to prevent detection bias.

#### Muscle size

**Figure 2:**
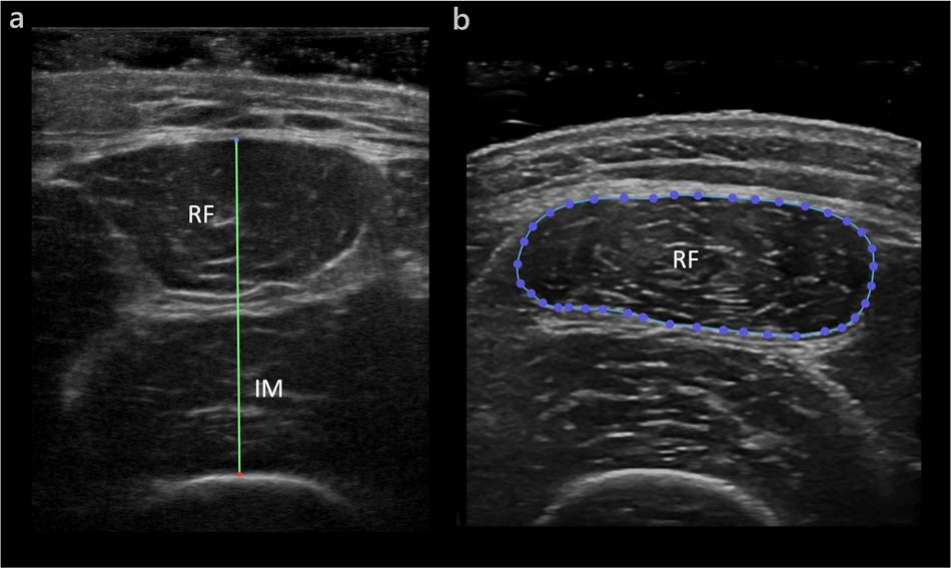
Example of ultrasound image analysis. (**a**) Quadriceps muscle layer thickness. (**b**) Cross-sectional area of the rectus femoris muscle. *RF* rectus femoris, *IM* intermedius. Adapted by Schieffelers DR *et al*. [61]. Copyright 2023 Elsevier

**Figure 3:**
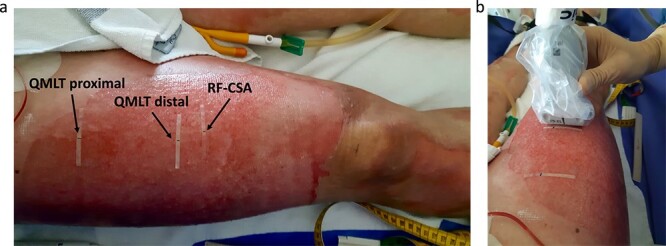
Ultrasound technique of quadriceps muscle size. (**a**) Measurement locations. (**b**) Sterile method over open wounds. *QMLT* quadriceps muscle layer thickness measured at the halfway point (= proximal) and two-thirds (= distal) between the anterior superior iliac spine and the superior pole of the patella, *RF-CSA* rectus femoris cross-sectional area as measured at the most proximal point where the rectus femoris muscle belly remained visible. Adapted by Schieffelers DR *et al*. [61]. Copyright 2023 Elsevier

B-mode ultrasound was used to determine quadriceps muscle layer thickness (QMLT) and rectus femoris cross-sectional area (RF-CSA), with QMLT as the primary outcome. QMLT comprises the combined thickness of the rectus femoris and intermedius muscle, measured between the superior fascial layer of the rectus femoris and the femoral periosteum [54] (Figure 2). Quadriceps ultrasound provides a valid and reliable measure to track muscle wasting and has been studied in various populations [55–60], including acute burn injuries [61]. To derive the QMLT, a multifrequency linear probe was aligned perpendicular to the longitudinal axis of the anterior thigh at the halfway and two-thirds point between the anterior superior iliac spine and the superior patellar pole [56] (Figure 3). RF-CSA was measured on the anterior thigh at the most proximal site where the rectus femoris muscle belly remained visible on the ultrasound screen. The ultrasound procedure was sterile in case the wound surface at the measurement points was not intact. Averages of triplicate bilateral measurements were calculated for both QMLT and RF-CSA, as this has been shown to reduce test–retest variability [61] and better reflect whole-body muscle mass [58,62]. Further details of the employed methods of quadriceps ultrasound have been described elsewhere [61]. Ultrasound data was exported and analyzed using DICOM reader software (Horos™ viewer v3.3.6, Horos Project).

#### Muscle force

**Figure 4:**
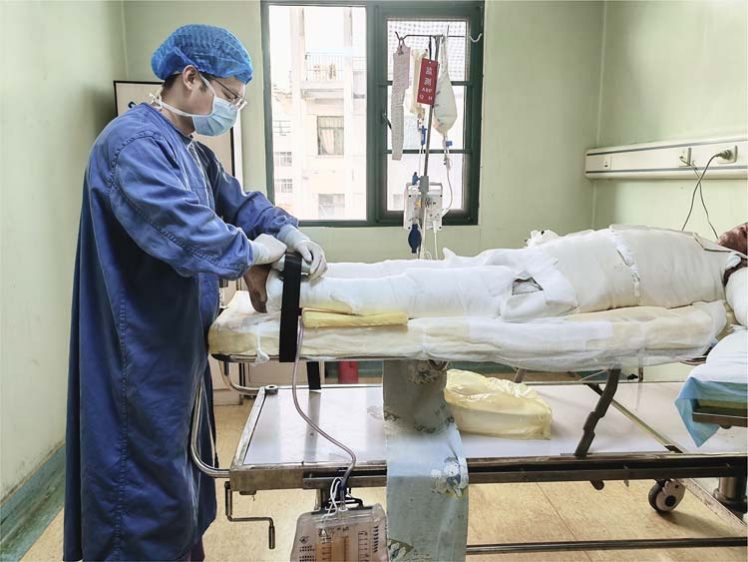
Muscle force assessment by hand-held dynamometry. A fixation band bound around the hospital bed provides counter-resistance while the assessor ensures correct positioning of the hand-held dynamometer

Handheld dynamometry (Lafayette, IN, USA) was used to measure changes in lower limb muscle force. Maximal voluntary contraction force of the lower limb was measured in the supine position with the dynamometer placed on the anterior surface of the distal tibia, just above the talocrural joint (Figure 4). Participants then carried out an isometric combined hip flexion and knee extension moment with both hip and knees extended to zero degrees. This muscle force measure is not a direct measure of quadriceps muscle force alone, but a compound measure of hip flexor and knee extensor muscles. A fixation band, fixed around the bed frame, provided the necessary counter-resistance to ensure isometric contraction during the test [63]. This test position was chosen to make muscle force assessment possible for bed-bound participants whose joint range of motion is too limited to reach traditional positions of muscle force assessment (e.g. 90° hip flexion and 90° knee flexion) due to bandages, pain and grafts interfering with joint movement in the context of acute burn wounds. As part of trial preparation this testing protocol displayed good reliability (intra-rater intraclass correlation coefficient (ICC) = −0.885, inter-rater ICC = −0.826; unpublished data in healthy adults). This data is in line with previously published reliability data of handheld dynamometry in burn patients [64]. The test maneuver was repeated three times with the highest value used for analysis. If the highest achieved force value was not within 10% of the second highest value, additional test maneuvers were carried out. Measurements where pain interfered with test validity were considered invalid if participants’ pain score on a 0–10 numeric rating scale was ≥6.

#### Quality of life

To capture the full impact of a burn injury on health-related quality of life, both generic and disease-specific data was collected using the Chinese language versions of the Burn Specific Health Scale Brief (BSHS-B) and the Eurocol Quality of Life-5 Dimensions (EQ-5D-5L) [65–67]. As the BSHS-B was primarily developed for the post-acute phase, not all the items were applicable during burn center stay and a total score could not be calculated. Instead, we report the subdomains ‘simple abilities’, ‘affect’ and ‘interpersonal relationships’ [66]. Items are scored on a five-point scale with lower scores indicating worse impact on quality of life [68]. The EQ-5D-5L entails rating the overall health state on a visual analogue scale of 0–100 (0 = worst possible health, 100 = best imaginable health) as well as scoring five dimensions (self-care, mobility, daily activities, pain/discomfort and anxiety/depression) on a five-point scale, roughly corresponding to no, slight, moderate, severe and extreme problems in ascending order. From each possible scoring combination in these five dimensions, the EQ-5D-5L utility index was derived based on a value set for Chinese urban residents [69]. This index ranges from −1 to 1, where 1 describes ‘full health’ and − 1 refers to the worst possible health status. Both the BSHS-B and EQ-5D-5L are validated questionnaires widely used in the burn population [70,71].

#### Feasibility and safety

Participants’ attendance rate in the exercise intervention was recorded, and reasons for non-attendance were noted. Furthermore, any adverse events occurring during or following exercise sessions were documented.

### Data collection

Data was collected by three assessors, with the goal of the same assessor performing follow-up assessments in the same participants. While therapists and assessors were not blinded to group assignment, they were blinded to results of the muscle-size assessment. This was achieved by pseudonymization of the exported ultrasound images, which were analyzed by a party without clinical involvement (DRS).

### Sample size

A target sample size of 58 participants was calculated using G*Power 3.1.9.2 based on a similar study by our group in Belgian burn centers [38]. To detect a group difference of 0.055 cm of QMLT with 80% power (alpha = 0.05, standard deviation = 0.073, effect size = 0.752), and taking an estimated dropout of 33% into account, 44 participants were required per study arm (n = 29 after dropout).

### Randomization

Following the baseline assessment (after eligibility screening and informed consent), participants were randomly allocated to either receive standard-of-care (control group) or standard-of-care and exercise training (exercise group). Participants were blinded to group allocation. A randomization sequence was generated using computerized minimization software (MinimPy) [72] with %TBSA (40–55, 56–70 or ≥70%) and age (18–41 or 42–64 years) as stratified factors and marginal balance as a distance measure to ensure group balance. Minimization is a dynamic randomization method by which group allocation is influenced by chance and existing group balance in the treatments and strata. By virtue of this method, the allocation sequence changed as the trial progressed, and was thereby concealed to all parties. To prevent further selection bias, input of new participants into the minimization matrix was completed by a party (DRS) blinded to participant characteristics other than %TBSA and age needed for stratification.

### Data analysis

Group comparisons of characteristics and baseline values of the study outcomes were performed using either independent t-tests, Mann–Whitney *U* test or Fisher’s exact test, as appropriate given the data type and normality. The effects of the experimental intervention on all dependent variables were tested using linear mixed models. Normality of the residuals was examined by histogram and homoscedasticity was checked by plotting the residuals vs. the predicted values. To account for individual participants’ change over time, the models employed subject ID as a random effect. Group type, weeks from baseline and their interaction effect (signifying the added effects of the experimental intervention) were used as fixed effects. The regression models furthermore controlled for a number of covariates, including %TBSA, age, gender, presence of inhalation trauma, postburn days until baseline, length of stay in the ICU, duration of mechanical ventilation and baseline values of study outcomes. These covariates were added by stepwise forward modeling to avoid multicollinearity as long as the two following conditions were met: (1) statistical significance of the respective covariate at *p* < 0.05 and (2) substantial model fit improvement as evaluated by a decrease of ≥10 points in the corrected Akaike information criterion [73]. Separate models were fitted for baseline to 6 weeks and from 6 to 12 weeks, as the assumption of linearity was violated for a combined model from baseline to 12 weeks for all outcomes. This study made use of an intention-to-treat analysis for missing data. For analysis of the muscle force values, models could only be fitted for the period between 6 to 12 weeks, as pain during the baseline assessment interfered with the validity of the muscle force assessment. Statistical significance was set at *p* < 0.05, and analysis was performed using JMP® Pro 17.0.0 (SAS Institute Inc., Marlow, UK).

## Results

### Participant flow

**Figure 5:**
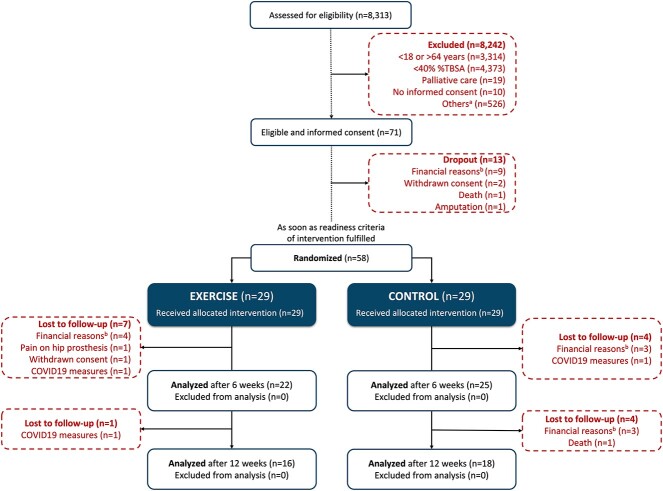
Study flow diagram. ^a^Others includes electrical burns, pregnancy, lower limb fractures or amputations, or any premorbid neurological, cardiovascular or psychological disorders expected to interfere with the intervention or outcome assessment. ^b^High out-of-pocket expenses associated with burn center stay resulting in early discharge

Participant flow throughout the study period is shown in Figure 5. Overall, formal informed consent was obtained from a total of 71 eligible participants upon admission to the burn center. Of these, 13 participants dropped out prior to the baseline assessment and random group allocation (early discharge due to financial reasons n = 9, withdrawn consent n = 2, death n = 1, amputation n = 1). The remaining 58 participants underwent random group allocation to the exercise (n = 29) or control (n = 29) group following the baseline assessment. Data from these 58 participants formed the basis for all reported baseline and outcome analysis. A total of 11 participants (exercise n = 7, control n = 4) dropped out between the baseline assessment and the 6-week assessment (financial reasons n = 7, COVID19-related measures n = 2, pain due to previous bilateral hip prosthesis n = 1, withdrawn consent n = 1), and a further 5 participants (exercise n = 1, control n = 4) dropped out between the 6 and 12 weeks assessment point (financial reasons n = 3, COVID19-related measures n = 1, death n = 1). The dropout of participants related to the COVID19 pandemic were the result of a shortage of rehabilitation staff due to state-enforced quarantine measures, rendering it impossible to continue study treatments.

### Characteristics

Clinical characteristics of the sample are presented in Table 1. Recruited patients were on average 42 [95% confidence interval (CI) 40–45] years old, had burns ranging from 40 to 94%TBSA [median 54%, interquartile range (IQR) 48–70%] of which almost all (median 95%, IQR 87–100%) were full thickness burns and were on their lower limbs (98%). No participants tested positive on daily nucleic acid testing for a SARS-COV2 infection during the study period. The majority of participants (86%) were mechanically ventilated following admission and 38% experienced septic episodes. All subjects underwent multiple grafting surgeries during the study period. Apart from the number of postburn days until baseline, clinical characteristics were comparable between groups. As such, the exercise group met the readiness criteria to start the experimental intervention 2 days earlier than the control group (7 vs. 9 days median, *p* = 0.045). With respect to baseline comparisons of outcomes, the exercise group presented with significantly larger baseline QMLT (*p* = 0.009). Groups were well-matched in all other outcomes at baseline (Table 1 and Table S1, see online supplementary material).

**Table 1:** Clinical characteristics. Postburn days until start of intervention refers to the point in time when participants met readiness criteria to start the assigned study intervention; normally distributed data is presented as mean and 95% CI; non-normally distributed data is presented as median and interquartile range

	**Exercise (29)**	**Control (29)**	***P*-value**
Gender (F/M)	7 /22	8 /21	0.764
Age, mean (95%CI)	43 (40–46) years	42 (38–46) years	0.581
TBSA, median [IQR] (range)	60% [45–67.5] (40–94)	50% [48–70] (40–80)	0.645
Full thickness, mean (95%CI)	54% (47–61)	51% (46–57)	0.501
Lower limb burns, n(%)	28 (97%)	29 (100%)	0.313
Burn type, n(%)			0.111
Flame	16 (55%)	23 (79%)	
Chemical	6 (21%)	5 (17%)	
Scald	5 (17%)	1 (3%)	
Contact	2 (7%)	0 (0%)	
Inhalation trauma	7 (24%)	6 (21%)	0.753
Duration mechanical ventilation, median (IQR)	4.5 (3–9.5) days	6 (4–9) days	0.618
Number of grafting surgeries, median (IQR)	4 (3–5)	4 (2.5–5)	0.269
Number of infections, median (IQR)	2 (1.25–4)	2 (1–3)	0.329
Number of septic episodes, median (IQR)	0 (0–2)	0 (0–1)	0.521
Revised BAUX score, mean (95%CI)	107 (97–117)	102 (95–109)	0.406
LOS in burn ICU, median (IQR)	7 (0–18) days	10 (0–12.8) days	0.969
LOS in burn center, median (IQR)	92 (61–110) days	91 (84–107) days	0.546
Postburn days until start of intervention, median (IQR)	7 (5–9) days	9 (6–12) days	0.045

*95%CI* 95% confidence interval, *IQR* interquartile range, *TBSA* total burn surface area, *LOS* length of stay, *ICU* intensive care unit, *F* female, *M* male

### Feasibility and safety

On average, participants in the exercise group followed 3.2 exercise sessions (95%CI 2.9–3.5) per week, with an attendance rate of 93% (95%CI 91–96). Grafting surgery and associated postsurgical bed rest were the main reason for not completing the exercise protocol, accounting for an average total of three sessions (95%CI 1.8–4.2) lost per participant. None of the exercise sessions were ceased prematurely due to pain. Similarly, participants did not require additional analgesia before, during or following exercise sessions. Few adverse events of the experimental intervention were observed. Four participants experienced postural hypotension during two to three sessions when transitioning from in-bed to out-of-bed exercises. Furthermore, participants in the exercise group developed blisters after wound closure more frequently than participants in the control group. Of all observed blisters, ~70% occurred in the exercise group and 30% in the control group and were more commonly located over joint surfaces where friction and pulling forces might have been causative factors. The majority of blisters were small, did not require deroofing and resolved spontaneously. Larger blisters were deroofed and covered with dressings. Blisters did not interfere with exercise training.

### Muscle size

**Figure 6:**
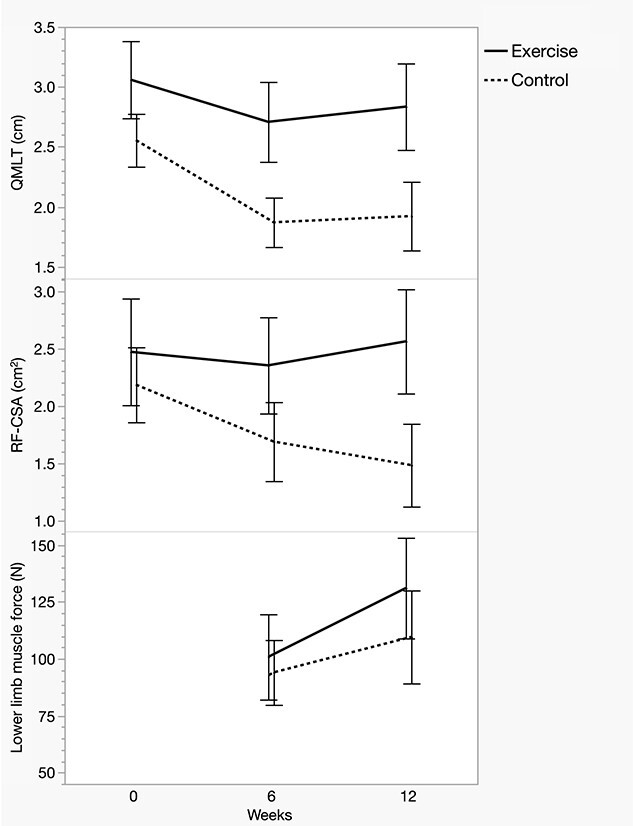
Group means for ultrasound-derived muscle size parameters and lower limb muscle force. Data presented as unadjusted means and error bars that depict 95% confidence intervals. Baseline values for lower limb muscle force are not shown as pain rendered the muscle assessment invalid at this time point. *N* Newton, *QMLT* quadriceps muscle layer thickness, *RF-CSA* rectus femoris cross-sectional area

Muscle size decreased from baseline to 6 weeks in both groups, with a more pronounced decrease in the control group (Figure 6). There was a significant interaction effect for exercise over time, with a mean retention of 0.306 cm of QMLT and 0.294 cm^2^ of RF-CSA from baseline to 6 weeks (Table 2). From 6 to 12 weeks of follow-up, the exercise group showed a faster recovery of QMLT (0.246 cm, *p* = 0.010) and RF-CSA (0.342 cm^2^, *p* < 0.001).

**Table 2:** Regression models for ultrasound-derived muscle size parameters, adjusted for baseline values. The significant ß-coefficient for interaction term ‘Group[Exercise]*Week’ signifies the added impact of the exercise intervention to standard care, expressed as absolute change per week of follow-up

		**Variable**	**β-Coefficient**	***P*-value**	**95%CI**	
**QMLT (cm)**	**0–6 weeks**	Group[Exercise]	0.056	0.461	−0.094	0.206
		Week	−0.117	<.001	−0.143	−0.092
		Group[Exercise]*Week	0.051	0.010	0.013	0.088
		Baseline value (0 weeks)	0.889	<.001	0.804	0.975
	**6–12 weeks**	Group[Exercise]	−0.193	0.176	−0.476	0.090
		Week	0.017	0.118	−0.004	0.038
		Group[Exercise]*Week	0.041	0.010	0.010	0.071
		Baseline value (6 weeks)	0.939	<.001	0.864	1.014
**RF-CSA (cm²)**	**0–6 weeks**	Group[Exercise]	0.029	0.753	−0.155	0.213
		Week	−0.080	<.001	−0.112	−0.048
		Group[Exercise]*Week	0.049	0.045	0.001	0.098
		Baseline value (0 weeks)	0.898	<.001	0.823	0.973
	**6–12 weeks**	Group[Exercise]	−0.336	0.021	−0.619	−0.054
		Week	0.004	0.718	−0.017	0.025
		Group[Exercise]*Week	0.057	<.001	0.026	0.088
		Baseline value (6 weeks)	0.996	<.001	0.933	1.059

*QMLT* quadriceps muscle layer thickness, *RF-CSA* rectus femoris cross-sectional area, *95%CI* 95% confidence interval

### Muscle force

Lower limb muscle force increased in both groups between 6 and 12 weeks of follow-up, with a significantly larger increase observed in the exercise group (interaction effect *p* < 0.001) (Table 3). The impact of exercise over this period increased the muscle force by 19 N, equivalent to a 19.5% improvement in group mean force values.

**Table 3:** Regression models for leg muscle force, adjusted for %TBSA. The significant ß-coefficient of interaction term ‘Group[Exercise]*Week’ signifies the added impact of the exercise intervention, expressed as absolute change of Newtons (N) per week of follow-up

		**Variable**	**β-Coefficient**	***P*-value**	**95%CI**	
**Lower limb muscle force (N)**	**0–6 weeks**	Group[Exercise]	−18.631	0.007	−31.971	−5.290
		Week	2.193	<.001	1.168	3.219
		Group[Exercise]*Week	3.102	<.001	1.604	4.601
		Baseline value (6 weeks)	1.002	<.001	0.942	1.063

*95%CI* 95% confidence interval

### Quality of life

**Figure 7:**
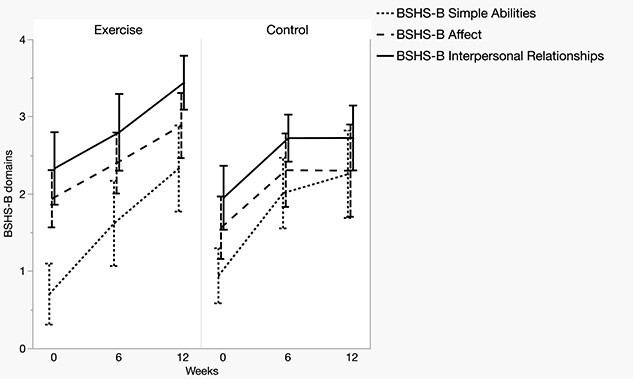
Group means for BSHS-B subdomains. Data presented as unadjusted means and error bars that depict 95% confidence intervals. *BSHS-B* Burn Specific Health Scale Brief

Baseline scores of all quality-of-life outcomes showed substantial impact of the burn injury on disease-specific and generic quality of life, with highly negative scores on the EQ-5D-5L health utility index, indicating health states perceived to be worse than death at baseline (Table S1, see online supplementary material). Both groups displayed comparable recovery trajectories over time in EQ-5D-5L parameters, with non-significant between-group differences (Table S2, see online supplementary material). With respect to the assessed BSHS-B subdomains, time trajectories of recovery were comparable between baseline and 6 weeks of follow-up; however, from 6 to 12 weeks the control group did not exhibit the continued improvement of the exercise group (Figure 7), with a significant interaction effect favoring the exercise group for the BSHS-B domains ‘affect’ (*p* = 0.022) and ‘interpersonal relationships’ (*p* = 0.040).

## Discussion

The main findings of this study indicate that early exercise training was associated with a protective effect on muscle size and force reduction during the first 6 weeks of exercise, and an improved recovery of muscle size and force in the second 6-week training period. No evidence of benefit for any of the assessed quality-of-life parameters could be found in the first 6-week training period, whereas in the second 6-week training period, early exercise training was associated with an improved recovery in the BSHS-B subdomains ‘affect’ and ‘interpersonal relationships’.

The administered training protocol succeeded in allowing participants to commence exercise at a crucial point in time (i.e. ~1 week after burn center admission). As such, our data shows that the negative impact of severe burn injury on muscle metabolism can be countered early on, as observed by a reduced loss of muscle size in the first 6 weeks of exercise. This is highly relevant as alterations in muscle metabolism are thought to be most prevalent in the first few weeks postburn when the hypermetabolic response reaches its peak [2,44,74]. Specifically, decreases in anabolic signaling (phosphoinositide 3-kinases-protein kinase B-mammalian target of rapamycin (PI3K–AKT–mTOR) pathway) have been shown to be dominant in the development of muscle wasting during the early postburn period [75]. As an anabolic stimulant, exercise might then have contributed to the observed retention of muscle size in this study [76,77]. Indeed, postburn exercise has been shown to stimulate the PI3K–AKT–mTOR pathway paralleled by improvements in muscle function [78]. Notably, while significant, our results may underestimate the true effect size of exercise training on muscle size in the first 6 weeks of exercise training. This is likely, because the exercise group presented with a significantly larger baseline muscle size than the control group. It has previously been observed that, in comparison to patients with smaller muscle size, patients with larger muscle size at admission experience relatively more muscle wasting over time [38,54]. Such an observation is likely the result of a more-to-give, more-to-lose paradigm, with larger muscles functioning as larger protein reserves that are more easily sacrificed as fuel for wound healing and increased energy demands. In contrast, patients with smaller muscle protein reserves reach a depleted status earlier, and consequently have less to spare. In that regard, maximizing the maintenance of muscle mass should be a therapy goal that is both desirable and acceptable during the acute phase of burns. This is especially relevant for wound healing, which depends on optimal macro- and micro-nutrient availability [79]. Together with adequate nutritional delivery, exercise training provides the anabolic stimulus that could increase the ability of hosts to metabolize ingested protein needed for wound healing [80,81]. Exercise training might also be able to improve the host’s capacity to utilize fat and carbohydrates, which could further positively impact wound healing [80,82].

Few studies have evaluated the effects of early exercise training on muscle wasting and muscle force. While our results corroborate findings of improved muscle mass and strength following exercise in animal burn models, and pediatric burns during the acute phase of burns and adult burns during later phases [83–86], they stand in contrast with findings from a similar study of adult burns by Gittings *et al*., who found no significant effects for fat-free mass or muscle force following 4 weeks of resistance training [33]. A plausible explanation for the contrast in findings is the difference in burn severity between recruited samples. Gittings *et al*. included non-severe burns between 5–40% TBSA (with the majority of patients at the lower end of burn severity) with a short burn center stay (median 12–13 days) [33]. Participants in the present study had severe burns ranging between 40–94% TBSA and spent a longer time in the burn center (median length of stay 91–92 days). Patients with minor burns might be overall less responsive to exercise as they likely undergo less muscle wasting and weakness than their severely burned peers. In addition, 4 weeks of exercise might not be long enough to elicit detectable changes in parameters of muscle mass and force. Indeed, a longer duration of early exercise training (up to 8 weeks) has been shown to exert a significantly higher retention of muscle size and force in adults with predominantly non-severe burns [38]. Despite the short exercise duration in the trial by Gittings *et al*., their exercise program had a positive impact on systemic inflammation [33]. This is noteworthy and highly desirable as inflammation is a significant contributor to postburn muscle wasting [87–89]. Furthermore, when comparing any effects, it needs to be considered that the study by Gittings *et al*. was not powered sufficiently to show equivalence in outcomes, thereby limiting the interpretability of their measured outcomes [33].

In regard to health-related quality of life, this study found no evidence of a significant influence of exercise training except for a significant improvement in the BSHS-B subdomains ‘affect’ and ‘interpersonal relationships’ between 6 and 12 weeks of follow-up. While outcome variability, limited sensitivity to change and insufficient statistical power limit conclusions, it seems plausible that any benefits of exercise would be more detectable at later stages of recovery—stages when the true impact of the burn injury will have had more time to transpire [71]. The finding that exercise training improved the BSHB-B subdomains ‘affect’ and ‘interpersonal relationships’ more than standard of care alone might be traced back to the fact that participation in the exercise protocol provided an opportunity to engage with other burn patients in the exercise room and overcome previous physical boundaries. Exercise training has previously been shown to have a positive impact on mental health [90]. Group exercise sessions might offer additional psychosocial benefits associated with increased peer support. This is particularly relevant given that family visits were entirely prohibited in line with COVID19-related measures. The observed lack of effects in the other assessed quality-of-life parameters is in line with previous reports of early exercise in adults [38,39] and children [91]. Besides being underpowered to detect effects, another methodological aspect that could explain the lack of efficacy in the quality-of-life domain is that our exercise training protocol was designed to achieve maximum metabolic modulation. This focus differs from more traditional burn rehabilitation protocols that have a primary focus on functional exercises [21]. Such exercises might be more applicable to patients’ quality of life. However, as postburn muscle wasting is commonly neglected as a metabolic sequela [21], we consider the lack of functional stimuli in our protocol an acceptable compromise. Lastly, as the administered training protocol in our study was challenging to many participants on multiple levels relating to their perceived health state (pain, anxiety, perceived safety, etc.), it is reassuring that we did not find any evidence of harm for the self-reported quality of life.

### Clinical relevance

This study identified several benefits of early exercise training that have significant implications for clinical practice. Given that postburn muscle wasting remains a challenge in burn care, the preservation and recovery of muscle mass and muscle function are highly desirable therapeutic targets, particularly following severe burn injuries. This is the first study to show beneficial adaptations in muscle size and force resulting from early exercise training in adults with severe burns. Incorporating exercise training as an adjunct to standard care during burn center stay resulted in an average weekly retention of 0.05 cm in QMLT and 0.05 cm^2^ in RF-CSA in the first 6 weeks. Over 6 weeks this equated to 11 and 13% additional retention of baseline value, respectively, amounting to 26% after 12 weeks, compared to standard care alone. Considering that a loss of 10% postburn muscle mass has been linked to complications such as impaired wound healing, insulin resistance and an increased risk of infections [12,13], such an improvement should be considered clinically significant. Over the duration of burn center stay such a cumulative effect might lead to a faster recovery, shorter length of stay and reduced health care expenses. However, as the length of stay in our study was highly confounded by the ability of participants to afford out-of-pocket expenses associated with specialized burn care in China (leading to early discharge) [92], such secondary outcomes remain to be tested. To prevent high out-of-pocket expenses, many burn patients in China (as is the case in many other regions [93]) choose to forego rehabilitation, seen as an optional luxury of burn care. Consequently, burn patients often develop preventable long-term disability which impedes return to work and reinforces cycles of socioeconomic disparities. By establishing evidence in support of the efficacy of burn rehabilitation, it is hoped that the results of this study will increase its perceived value and encourage its adoption into clinical practice [94].

Widespread inconsistencies in the use of exercise training during burn center stay have hindered its implementation [92,95,96]. The fact that burn patients and clinicians often perceive exercise training during the acute phase of burns as highly challenging is certainly a contributing factor [97]. The present study supports the overall safety and feasibility of exercise training commenced shortly after burn center admission. The higher incidence of postburn blistering in the exercise group was not interpreted as a serious complication, given that blistering is a common transient occurrence during burn recovery that mostly resolves spontaneously [98]. However, this finding highlights the need for therapists to be vigilant in regards to monitoring and addressing any blisters to avoid potential sequelae. Blistering might indirectly exert a potential negative impact on scar formation by interfering with the use of compression garments and causing prolonged healing. While it did not affect the use compression garments in our study, further study of the effect of exercise-induced blisters on scar quality is needed to establish this as a complication. Underlying the multi-disciplinary decision whether or not exercise training is provided to severely burned patients is a cost–benefit analysis. The results of this study emphasize several important benefits of early exercise training while acknowledging the potential for minor adverse events.

This study also underlines the importance of a concerted effort of the multi-disciplinary team to achieve early exercise participation. In many high-income regions, patients with severe burn injuries are often sedated and mechanically ventilated for prolonged periods of time [99]—a practice that interferes with exercise participation [97]. It is of interest that the duration of mechanical ventilation in our sample, on average 4 to 6 days, was far lower than has been reported in other regions [99], pointing to the potential of early extubation to facilitate early exercise [100,101].

### Strengths and limitations

There is currently little evidence surrounding exercise training during burn center stay in severely burned adults. A major reason for this certainly relates to the fact that severe burns are less common in regions where most of the research funding exists and where rehabilitation is most established. In light of this, a main strength of this study is that it recruited participants from the largest burn center in China, where, similar to other low-/middle-income countries, severe burn injuries are more common and rehabilitation is still considered a young profession [92,94]. With >5000 admissions annually, the department of burns in Wuhan serves a population of almost 60 million as the only burn center in the province of Hubei [102]. The present study currently forms the first and largest clinical trial to date incorporating resistance and aerobic training in severely burned adults commenced during the acute phase of burns. Another strength of this study pertains to the use of ultrasound to quantify muscle size—a tool with good clinical applicability to monitor postburn muscle wasting at all stages of burn recovery [61], which is particularly relevant given that muscle wasting is not commonly measured in burn care and intervention trials [21,103].

Several limitations need to be considered when interpreting the findings of this study. Firstly, it is important to note that the comparator group in our study followed a relatively passive standard-of-care treatment that did not include ambulation and aerobic or resistance training. While this affects the applicability of our findings to settings with a more active standard-of-care, it supports keeping resistance and aerobic training as part of the standard-of-care, if already provided, or adding it if not yet included. A second limitation relates to potential performance and detection bias in our study, as is common in non-pharmacological trials [104]. Although it is our view that blinding of participants was successfully achieved, we cannot rule out that participants became aware of which group they were allocated to as no placebo intervention was provided to participants in the control group. However, none of the participants indicated knowledge of group belonging throughout the study duration. Likewise, we were unable to blind assessors, as the staff that carried out the outcome assessment also administered the intervention. However, the potential of detection bias was minimized by (1) blinding assessors to the results of all previous outcome assessment, and (2) conducting the ultrasound image measurement of quadriceps muscle size, as the primary outcome in this study, by a party without clinical involvement (DRS). Lastly, it needs to be noted that the random allocation process failed to achieve group balance in the QMLT value at baseline, burn type and ICU length of stay. Although the latter two were not significantly different between groups and the former was controlled for in the statistical analysis, the impact of these variables needs to be considered.

### Future directions

The positive impact of early exercise training on muscle outcomes, as shown in this study, leads to further questions about the underlying mechanisms. Understanding exercise-induced changes in systemic inflammation, hyperglycemia and insulin sensitivity as well as anabolic and anti-catabolic actions will aid in optimizing exercise training delivery to better target postburn muscle wasting and related metabolic morbidity. Another area of particular interest for low-/middle-income regions where financial access to exercise rehabilitation presents a major barrier, is its cost-effectiveness. Hospital length of stay, days until wound closure and time till return to work relative to measures of disease burden are associated measures relevant to burn survivors and their families [105]. Future study designs should incorporate different experimental groups, comparing different exercise programs and starting points (early vs. late), and include long-term follow-up beyond discharge where possible. Lastly, the elderly burn population is a challenging age group with prolonged convalescence and metabolic morbidity [45,46], that while excluded in this study to reduce sample heterogeneity, represents a particular group that may benefit from the early institution of exercise training. In regards to this age group, it needs to be kept in mind that the age of 65 years likely does not represent a physiological cut-off point for differing responses to burn injury and exercise.

## Conclusions

The evidence provided by this study supports the inclusion of exercise training into the acute management of adults with severe burn injury. Early exercise training is a safe and efficacious strategy that appears to promote the retention and recovery of postburn muscle size and muscle strength.

## Supplementary Material

Supplementary_material_(new)_tkae005

## Data Availability

All data collected for this study can be obtained by correspondence with the corresponding authors.

## Abbreviations

BSHS-B: Burn Specific Health Scale Brief; CI: Confidence interval; EQ-5D-5L: Eurocol Quality of Life-5 Dimensions; ICU: Intensive care unit; IQR: Interquartile range; QMLT: Quadriceps muscle layer thickness; RF-CSA: Rectus femoris cross-sectional area; TBSA: total burn surface area.
