# Supplementary material for: Effects of early exercise training following severe burn injury: a randomized controlled trial
Source: Burns Trauma. 2024 May 7;12:tkae005. doi: 10.1093/burnst/tkae005 (PMC11075770; doi:10.1093/burnst/tkae005)
Supplement: Supplementary_material_(new)_tkae005 [file supplementary_material_(new)_tkae005.docx]

**Supplementary material**

**Table S1. Group means per outcome and time points**

|  | **Weeks** | **n** | **Exercise** | **n** | **Control** |
| --- | --- | --- | --- | --- | --- |
| **QMLT** (cm) | 0 | 23 | 3.060 [2.738;3.382] | 25 | 2.554 [2.334;2.775] |
|  | 6 | 20 | 2.708 [2.374;3.041] | 23 | 1.869 [1.663;2.075] |
|  | 12 | 15 | 2.835 [2.474;3.195] | 16 | 1.921 [1.635;2.208] |
| **RF-CSA** (cm^2^) | 0 | 21 | 2.471 [2.006;2.936] | 24 | 2.184 [1.858;2.511] |
|  | 6 | 18 | 2.353 [1.934;2.773] | 22 | 1.689 [1.345;2.033] |
|  | 12 | 14 | 2.562 [2.108;3.017] | 16 | 1.484 [1.123;1.845] |
| **Lower limb muscle force (N)** | 0 | - | N.A. | - | N.A. |
|  | 6 | 22 | 100.793 [82.043;119.543] | 22 | 93.996 [79.761;108.231] |
|  | 12 | 15 | 131.043 [108.920;153.167] | 17 | 109.582 [89.150;130.015] |
| **EQ-5D-5L health utility index** | 0 | 29 | -0.286 [-0.341;-0.232] | 29 | -0.317 [-0.362;-0.271] |
|  | 6 | 22 | -0.076 [-0.234;0.083] | 25 | 0.037 [-0.149;0.224] |
|  | 12 | 16 | 0.273 [0.113;0.434] | 18 | 0.292 [0.096;0.487] |
| **EQ-5D-5L VAS** | 0 | 29 | 27.293 [20.267;34.319] | 28 | 27.179 [19.686;34.671] |
|  | 6 | 22 | 39.795 [31.044;48.547] | 24 | 41.042 [30.537;51.547] |
|  | 12 | 16 | 54.563 [42.362;66.763] | 18 | 54.361 [43.961;64.761] |
| **BSHS-B simple abilities** | 0 | 29 | 0.701 [0.306;1.096] | 29 | 0.939 [0.582;1.295] |
|  | 6 | 22 | 1.616 [1.064;2.168] | 25 | 2.009 [1.552;2.466] |
|  | 12 | 16 | 2.326 [1.769;2.884] | 18 | 2.253 [1.687;2.819] |
| **BSHS-B affect** | 0 | 29 | 1.935 [1.565;2.306] | 29 | 1.560 [1.156;1.965] |
|  | 6 | 22 | 2.398 [2.003;2.792] | 25 | 2.305 [1.829;2.781] |
|  | 12 | 16 | 2.883 [2.461;3.304] | 18 | 2.299 [1.701;2.896] |
| **BSHS-B interpersonal relationships** | 0 | 29 | 2.328 [1.858;2.797] | 29 | 1.948 [1.534;2.362] |
|  | 6 | 22 | 2.795 [2.299;3.292] | 25 | 2.720 [2.416;3.024] |
|  | 12 | 16 | 3.438 [3.088;3.787] | 18 | 2.722 [2.302;3.142] |

Data presented as unadjusted means with 95% confidence intervals

*QMLT* quadriceps muscle layer thickness, *RF-CSA* rectus femoris cross-sectional area, *EQ-5D-5L* Eurocol Quality of Life-5 Dimensions, *BSHS-B* Burn Specific Health Scale Brief, *VAS* Visual Analogue Scale

**Table S2. Regression models for quality-of-life measures, adjusted for covariates**

|  |  | **Variable** | | **β-coeff.** | ***p*-value** | **95%CI** | |
| --- | --- | --- | --- | --- | --- | --- | --- |
| **EQ-5D-5L Health Utility Index** | **0-6 weeks** | | Group[Exercise] | -0.004 | 0.957 | -0.136 | 0.128 |
|  |  |  | Week | 0.062 | <.001 | 0.039 | 0.085 |
|  |  |  | Group[Exercise]*Week | -0.025 | 0.133 | -0.058 | 0.008 |
|  |  |  | Baseline value (0 weeks) | 1.120 | <.001 | 0.736 | 1.504 |
|  | **6-12 weeks** | | Group[Exercise] | -0.118 | 0.432 | -0.415 | 0.180 |
|  |  |  | Week | 0.050 | <0.001 | 0.028 | 0.073 |
|  |  |  | Group[Exercise]*Week | 0.017 | 0.313 | -0.016 | 0.050 |
|  |  |  | Baseline value (6 weeks) | 0.853 | <0.001 | 0.728 | 0.978 |
| **EQ-5D-5L**  **VAS** | **0-6 weeks** | | Group[Exercise] | 0.458 | 0.885 | -5.822 | 6.738 |
|  |  |  | Week | 2.837 | <.001 | 1.720 | 3.954 |
|  |  |  | Group[Exercise]*Week | -0.277 | 0.728 | -1.865 | 1.311 |
|  |  |  | Baseline value (0 weeks) | 0.933 | <.001 | 0.800 | 1.066 |
|  | **6-12 weeks** | | Group[Exercise] | -0.982 | 0.908 | -17.944 | 15.980 |
|  |  |  | Week | 2.424 | <0.001 | 1.125 | 3.722 |
|  |  |  | Group[Exercise]*Week | 0.141 | 0.881 | -1.744 | 2.027 |
|  |  |  | Baseline value (6 weeks) | 0.893 | <0.001 | 0.759 | 1.026 |
| **BSHS-B Simple Abilities** | **0-6 weeks** | | Group[Exercise] | -0.136 | 0.599 | -0.647 | 0.376 |
|  |  |  | Week | 0.185 | <.001 | 0.134 | 0.235 |
|  |  |  | Group[Exercise]*Week | -0.025 | 0.498 | -0.098 | 0.049 |
|  |  | | R-BEAUX Score | -0.021 | <.001 | -0.032 | -0.010 |
|  | **6-12 weeks** | | Group[Exercise] | -0.569 | 0.211 | -1.469 | 0.332 |
|  |  |  | Week | 0.070 | 0.046 | 0.001 | 0.139 |
|  |  |  | Group[Exercise]*Week | 0.085 | 0.097 | -0.016 | 0.185 |
|  |  |  | Baseline value (6 weeks) | 0.843 | <0.001 | 0.715 | 0.971 |
| **BSHS-B Affect** | **0-6 weeks** | | Group[Exercise] | 0.113 | 0.467 | -0.195 | 0.421 |
|  |  |  | Week | 0.142 | <.001 | 0.088 | 0.196 |
|  |  |  | Group[Exercise]*Week | -0.078 | 0.053 | -0.156 | 0.001 |
|  |  |  | Baseline value (0 weeks) | 0.808 | <.001 | 0.708 | 0.909 |
|  |  | | Duration on mechanical ventilation | -0.026 | 0.007 | -0.045 | -0.007 |
|  | **6-12 weeks** | | Group[Exercise] | -0.480 | 0.124 | -1.096 | 0.137 |
|  |  |  | Week | 0.021 | 0.368 | -0.026 | 0.068 |
|  |  |  | Group[Exercise]*Week | 0.081 | 0.022 | 0.012 | 0.150 |
|  |  |  | Baseline value (6 weeks) | 0.926 | <0.001 | 0.824 | 1.027 |
| **BSHS-B Interpersonal Relationships** | **0-6 weeks** | | Group[Exercise] | 0.379 | 0.182 | -0.181 | 0.940 |
|  |  |  | Week | 0.134 | <.001 | 0.059 | 0.208 |
|  |  |  | Group[Exercise]*Week | -0.057 | 0.295 | -0.164 | 0.051 |
|  | **6-12 weeks** | | Group[Exercise] | -0.560 | 0.179 | -1.484 | 0.284 |
|  |  |  | Week | 0.016 | 0.641 | -0.052 | 0.084 |
|  |  |  | Group[Exercise]*Week | 0.104 | 0.040 | 0.005 | 0.203 |
|  |  |  | Baseline value (6 weeks) | 0.661 | <0.001 | 0.535 | 0.786 |

The significant ß-coefficient of interaction term “Group[Exercise]*Week” signifies the added impact of the exercise intervention, expressed as absolute change per week of follow-up

*EQ-5D-5L* Eurocol Quality of Life-5 Dimensions, *BSHS-B* Burn Specific Health Scale Brief, *VAS* Visual Analogue Scale
